# O076: Impact of the implementation of the who hand hygiene promotion strategy in the Hong Kong pilot site: 2006-2012

**DOI:** 10.1186/2047-2994-2-S1-O76

**Published:** 2013-06-20

**Authors:** JWM Tai, D Pittet, WH Seto, V Cheng, P Ching

**Affiliations:** 1 Infection Control, Queen Mary Hospital, Hong Kong, China; 2 Infection Control, University of Geneva Hospitals, Geneva, Switzerland; 3 WHO Collaborating Centre, Hong Kong, Hong Kong; 4 Queen Mary Hospital, Hong Kong, Hong Kong; 5 WHO Collaborating Centre, Hong Kong, Hong Kong

## Introduction

The WHO First Global Patient Safety Challenge “Clean Care is Safer Care” aims to tackle healthcare-associated infection worldwide with hand hygiene (HH) as the cornerstone intervention.

## Objectives

To assess the impact and sustainability of the WHO hand hygiene multimodal improvement strategy in the Hong Kong pilot site hospital.

## Methods

Times series design with experimental and control wards. Data were collected during three cycles of action research targeted at improving compliance with practices.

## Results

Overall hand hygiene compliance was 22% (experimental wards, 18.3%; controls, 25.4%) at baseline. At the end of the first action research cycle (April–Dec 2006), compliance improved to 41.6% in experimental wards and decreased to 18.8% in control wards. When multimodal interventions were extended to six study sites in the second action research cycle (Jan–May 2007), experimental wards showed sustainability (44.4%), while no change was observed in control wards (25.6%). During the last action research cycle (May 07– Feb 2008), overall compliance increased to 54% (experimental wards, 52.6%; controls, 55.3%). Further reinforcement strategies were implemented from March 2008 and overall hospital compliance increased to 78.6% in 2012. In parallel, the use of alcohol-based handrub increased from 2L/1000 patient-days in 2006 to 45L/1000 patient-days in 2012. Methicillin-resistant *Staphylococcus aureus* bloodstream infection decreased from 1.53/1’000 patient-days in 2006 to 0.87/1’000 patient-days in 2012.

## Conclusion

The WHO HH promotion strategy, using an action research approach, is successful with sustained compliance and continuous reduction in MRSA bloodstream infection rates.

## Disclosure of interest

None declared.

